# Effects of placebo on bench throw performance of Paralympic weightlifting athletes: a pilot study

**DOI:** 10.1186/s12970-019-0276-9

**Published:** 2019-02-19

**Authors:** Gustavo De Conti Teixeira Costa, Luan Galvão, Martim Bottaro, João Felipe Mota, Gustavo Duarte Pimentel, Paulo Gentil

**Affiliations:** 1 Faculdade de Educação Física e Dança, Universidade Federal de Goiás, Goiás, Brazil; 2 Faculdade de Educação Física, Universidade de Brasília, Distrito Federal, Brasília, Brazil; 3 Faculdade de Nutrição, Universidade Federal de Goiás, Goiás, Brazil

**Keywords:** Nutritional supplements, Sports performance, Psyching up

## Abstract

**Background:**

The aim of the present study was to analyse the effects of placebo on bench throw performance in Paralympic weightlifting athletes.

**Methods:**

The study involved four Paralympic weightlifting male athletes (age: 40.25 ± 9.91 years, weight: 60.5 ± 8.29 kg, height: 1.60 ± 0.15 m) that visited the laboratory in three occasions, separated by 72 h. In the first session, the athletes were tested for bench press one repetition maximum (1RM). The other two sessions were performed in a randomized counter-balanced order and involved bench throw tests performed either after taking placebo while being informed that the capsule contained caffeine or without taking any substance (control). The bench throw tests were performed with loads corresponding to 50, 60, 70 and 80% of the bench press 1RM.

**Results:**

According to the results, mean velocity (∆: 0.08 m/s, ES 0.36, *p* < 0.05) and mean propulsive velocity (∆: 0.11 m/s, ES 0.49, *p* < 0.05) at 50% of 1RM were significantly higher during placebo than control (*p* < 0.05). However, there were no difference between control and placebo for 60, 70 and 80% of 1RM (*p* > 0.05).

**Conclusion:**

Our results suggest that placebo intake, when the athletes were informed they were taking caffeine, might be an efficient strategy to improve the performance of explosive movements in Paralympic weightlifting athletes when using low-loads. This brings the possibility of using placebo in order to increase performance, which might reduce the risks associated with ergogenic aids, such as side-effects and positive doping testing.

## Background

The intake of nutritional supplements are a common practice among physical activity practitioners and athletes; however, few actually have scientific evidence for their efficacy [1]. Among them, caffeine is one of the most consumed substances by athletes [2] and is considered to positively impact physical performance [3]. In this regard, previous studies suggested that caffeine may be ergogenic, sparing muscle glycogen improving pain tolerance, reducing rate of perceived exertion, increase maximum voluntary contraction, strength and power in high-intensity activities besides of stimulating central nervous system [4–10]. However, there are specific controversies about its effects, mainly when the studies compared the acute effects of caffeine vs placebo intake showed inconsistent conclusions [11–17].

While genetic factors might explain a large portion of the variance associated with the caffeine effects such as pain tolerance, anxiogenic and alert effects [18–20], there are important psychological responses to ingesting a substance that should be considered. The placebo effect [21] can influence the physiological aspects to physical exercise performance [3]^,^ one of the all factors that might influence the effects of caffeine is the placebo effect [22]. In agreement with this, Saunders et al. [23] found an improvement in the cycling time to exhaustion in trained cyclists who ingested placebo believing to have ingested caffeine. Similarly, in a previous study, Beedie et al. [24] reported that when competitive male cyclists ingested placebo believing to have ingested caffeine there were increases in aerobic power at VO2max test and 10-km time trials, with no difference in oxygen uptake, heart rate, and blood lactate. Such placebo effects do not rule out a true effect of caffeine supplementation. Although these studies suggested that aerobic performance improves following placebo intake when individuals believed that they are ingesting caffeine, we are not aware of studies that measured the placebo effect in powerlifting. Moreover, Paralympic athletes have been shown to have psychological particularities that might make them especially vulnerable to the placebo effect, such as, concerns about having to perform consistently well throughout training and difficulties in coping with negative results [25]. Therefore, it seems important to perform specific studies in these group despite the difficulty to select subjects with these characteristics.

The analysis of the placebo effect might be of great practical importance since it could provide an alternative for improving performance through the intake of an inert substance, with no risk of testing positive for doping or adverse effects, either due to the direct use of prohibited substances, or by the possible contamination of nutritional supplements [10, 26–30]. Based on this, the present study aimed to evaluate if muscle performance during explosive movements would change in Paralympic weightlifting athletes after the intake of placebo when the participants were informed that they were taking caffeine.

## Materials and methods

### Participants

Four Paralympic weightlifting male athletes were recruited to participate in this study (age: 40.25 ± 9.91 y, weight: 60.5 ± 8.29 kg, height: 1.60 ± 0.15 m). One had dwarfism, one myelomeningocele and hydrocephalus and two poliomyelitis. Athletes trained regularly five times a week aiming to compete, and all had previous experience with caffeine use, but have not taken any caffeine supplements in the previous six months. Two athletes had won medals in at least two phases on the national circuit. They were only allowed to participate if they had no orthopaedic or cardiometabolic problems that could be aggravated by the study protocol, as attested by a physician. This study was approved by Federal University of Goias committee (2.058.322) and all the participants signed a written informed consent form before participation (Table 1).

**Table 1:** Characteristics of the participants

	Mean	Minimum	Maximum
Age (years)	40.25 ± 9.91	26	54
Weight (kg)	60.6 ± 8.36	49.0	71.2
Height (m)	1.61 ± 0.16	1.36	1.78
Body mass index (kg/m^2^)	23.83 ± 4.48	19.38	30.82
1 repetition maximum load (kg)	69 ± 19.46	40	92

### Procedures

The study is a randomized, double-blind, crossover study. The athletes attended the laboratory three times, with an interval of 72 h between visits. During the first visit, they were submitted to the one repetition maximum test (1RM) in the bench press, as previously recommended [31]. The second and third visits involved the bench throw tests. The athletes were randomly assigned to ingest a capsule of placebo or no capsule on the second and third visits, in a cross-over design. During the placebo situations, the athletes received one capsule containing maize starch one-hour prior the test and were informed that it contained 6 mg.kg-1 caffeine. The athletes were oriented to avoid caffeine containing beverages and foods one week prior to the beginning of the study.

### Data collection

Muscular performance was measured in the bench throw, using an isoinertial indicator (T-Force, Dynamic Measurement System; Ergotech Consulting S.L., Murcia, Spain). The exercise was performed on a smith machine and the athletes were instructed to perform three repetitions with maximum intended velocity in all repetitions. The tests were performed with 50, 60, 70 and 80% of 1RM, with 5 min of rest between each load condition. The movement started with elbows fully extended and then the bar was get down until touching the sternum. A linear position transducer was attached to the bar. The bar position data were sampled at 1000 Hz using a computer, as recommended by the manufacturer. The finite differentiation technique was used to calculate the velocity and acceleration of the bar, presenting an associated error of < 0.25%, while the displacement was accurate to ±0.5 mm [32].

### Statistical analysis

Data were analysed using the Statistical Package of Social Science software (SPSS 20.0, Chicago, IL, USA). Factorial ANOVA with a within-within design was used to compare the performance between placebo vs control situation at different loads. When necessary multiple comparisons were used as post hoc. Data were considered statistically significant when *p* < 0.05. Effect size (ES) of the mean differences was determined using Cohen’s d. The magnitude of the ES was determined by Hopkin’s scale as follows: < 0.1 (trivial), 0.1–0.3 (small), 0.3–0.5 (moderate), 0.5–0.7 (large), 0.7–0.9 (very large) and > 0.9 (perfect) [33].

## Results

**Fig. 1:**
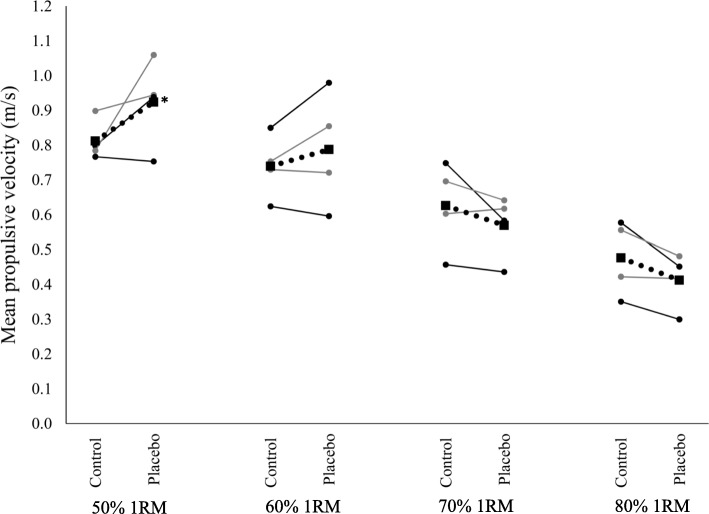
Mean propulsive velocity (m/s)

Table 2 shows the results relative to the absolute velocity values ​​as a function of the load lifted. Although the mean velocity to peak and peak velocity were not different between situations, the mean velocity at 50% RM was significantly higher in placebo vs control (∆: 0.08 m/s), with moderate effect size (0.36; *p* < 0.05). Similarly, mean propulsive velocity at 50% RM was significantly higher in placebo vs. control (∆: 0.11 m/s) with moderate effect size (0.49; *p* < 0.05) as shown in Fig. 1.

**Table 2:** Comparison between the placebo and control group on velocity of displacement of the bar in bench press

Variable		50% 1RM	ES	60% 1RM	ES	70% 1RM	ES	80% 1RM	ES
Mean velocity (m/s)	Control	0.76 ± 0.08	0.36*	0.70 ± 0.07	0.19	0.61 ± 0.10	0.26	0.47 ± 0.10	0.31
	Placebo	0.84 ± 0.12		0.74 ± 0.12		0.56 ± 0.08		0.41 ± 0.08	
Mean velocity to peak (m/s)	Control	0.79 ± 0.09	0.33	0.72 ± 0.07	0.18	0.61 ± 0.11	0.19	0.48 ± 0.10	0.31
	Placebo	0.87 ± 0.13		0.76 ± 0.13		0.57 ± 0.09		0.42 ± 0.08	
Mean propulsive velocity (m/s)	Control	0.81 ± 0.09	0.46*	0.74 ± 0.09	0.19	0.63 ± 0.12	0.27	0.48 ± 0.10	0.36
	Placebo	0.92 ± 0.12		0.79 ± 0.15		0.57 ± 0.09		0.41 ± 0.08	
Peak velocity (m/s)	Control	1.16 ± 0.11	0.37	1.06 ± 0.13	0.14	0.91 ± 0.19	0.27	0.72 ± 0.19	0.32
	Placebo	1.27 ± 0.16		1.11 ± 0.21		0.82 ± 0.11		0.61 ± 0.12	

*ES* effect size, *RM* Repetition Maximum

*significant difference between placebo and control (*p* < 0,05)

## Discussion

To the best of our knowledge, this was the first study to analyse the placebo effects of caffeine on bench throw performance of Paralympic weightlifting athletes. According to the results, the ingestion of placebo significantly increases mean velocity and mean propulsive velocity at 50% of 1RM. These findings may be particularly interesting, since this intensity is in the recommend range for maximum power output in the bench throw [34]. Moreover, considering that the tests involved highly trained athletes, the differences might be relevant to training and competition. It is important to note that, whilst they did not reach significance, the differences with higher loads occurred in the opposite direction, with a trend for a detrimental effect with placebo. The reason for this is not known, but there are two hypotheses to consider. First, the tests were incremental, so it might be possible that the higher performance in the earlier sets with lower loads lead to fatigue in the later sets, performed with higher loads. Second, when the load increased and becomes more challenging, the participants might have expected to have an improved performance perform. However, since there was not physiologic enhancement due to the supplementation, a negative psychological influence might have occurred.

Although the deception used in the present study is not common in scientific literature, it is closer to what happens in real world, where many athletes take nutritional supplements believing on a true physiological effect, which might affect the results [22]. Besides that, uptake placebo was able to improve performance on bench throw with no reports of adverse effects already shown to caffeine supplementation before [10]. In this regard, Hurst et al. suggested that the intention to improve performance by the athletes when taking placebo can make a difference in the final performance; therefore, in order to take full advantage of this intervention, the athletes should believe in the benefits of the ingested substance [35].

Whilst many athletes believe that nutritional supplements are related to performance enhancements [36]^,^ most do not obtain adequate information and do not even know the active ingredients or mechanism of action of the substances used [36–42]. Therefore, supplements use seems to rely more on beliefs than on scientific evidence. Considering our findings that an inert substance might increase performance when athletes were deceived to believe it was an ergogenic aid; this might help to explain the divergence that often occurs between anecdotal and scientific evidence. On the other hand, this study had the focus only in placebo effect and a third group of caffeine was not used aiming to compare three groups.

Regarding the possible explanation for the placebo effect, Beedie et al. divided it in four categories: pain reduction, belief-behaviour relation, attentional changes and arousal changes. Within these mechanisms, the improvements found in our study can be explained by attentional and arousal changes [24]. Besides that, caffeine intake also increasing pain tolerance [43], however, this probably did not happen in this study due to short duration with just three repetitions of exercise performed. The placebo effect might be associated with self-directed cognitive strategies and preparatory arousal (i.e. including imagery and attentional focus), which has been shown to enhance force production [44, 45].

The major limitation of the present study is the low number of participants. However, due to the characteristics of the participants, it would be difficult to obtain a higher sample size. It would also be interesting to have a third situation, with caffeine use. Future research should be conducted on a higher number of athletes, including Paralympic athletes with different limitations. Moreover, it would be valuable to assess the long-term effects of placebo, in order to test if the regular increase in performance over training sessions might bring long-term benefits.

## Conclusions

Our results suggest that placebo intake, when the athletes believe they are taking caffeine, might be an efficient strategy to improve performance in the bench throw test in Paralympic weightlifting athletes under low-loads. This brings the possibility of using placebo in order to increase performance in plyometric and speed exercises, reducing the side effects and risks associated with the use of ergogenic aids. Additionally, it would be ideal that nutritional strategy was investigated with high loads before using it in practice. Finally, it might be suggested that part of the conflict that usually exists between anecdotal reports and scientific evidence about nutritional supplementation can be associated to the psychological effects of ingesting a supplement.
